# Enhanced Sampling and Tailored Collective Variables Yield Reproducible Free Energy Landscapes of Beta‑1 Adrenergic Receptor Activation

**DOI:** 10.1021/acs.jctc.5c00600

**Published:** 2025-07-28

**Authors:** Simone Aureli, Valerio Rizzi, Nicola Piasentin, Francesco Luigi Gervasio

**Affiliations:** † School of Pharmaceutical Sciences, 27212 University of Geneva, Rue Michel-Servet 1, CH-1206 Geneva, Switzerland; ‡ Institute of Pharmaceutical Sciences of Western Switzerland, 27212 University of Geneva, CH-1206 Geneva, Switzerland; § Swiss Bioinformatics Institute, 27212 University of Geneva, CH-1206 Geneva, Switzerland; ∥ Chemistry Department, University College London (UCL), WC1E 6BT London, U.K.

## Abstract

The beta-1 adrenergic receptor (ADRB1) is a critical target for cardiovascular drugs, yet our understanding of how it is activated remains incomplete. Capturing the concerted interplay of agonists, solvent, ions, and protein microswitches is a significant challenge for conventional simulation methods and is essential for unraveling this process. Here, we address this challenge by implementing a powerful enhanced sampling framework that integrates the OneOPES enhanced sampling algorithm with a set of biologically motivated collective variables (CVs). These CVs are designed to track several key features of the activation process simultaneously, including rearrangement of conserved microswitches, the state of the sodium ion binding pocket, and dynamics of critical water molecules. Using this framework, we mapped the multidimensional free energy landscapes of the ADRB1 receptor in both its apo- and adrenaline-bound holo states. Our analysis reveals a detailed, stepwise activation pathway that quantifies the known modulatory roles of sodium ions and protonation states and identifies essential water-mediated networks that stabilize the active conformation. This work provides a detailed overview of ADRB1 activation and establishes the robustness of our OneOPES approach for investigating complex activation mechanisms with the potential for application to other Class A GPCRs.

## Graphical abstract

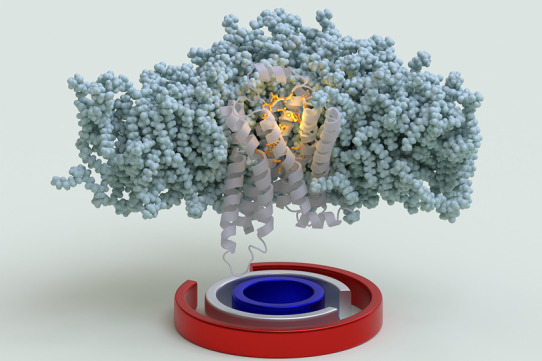

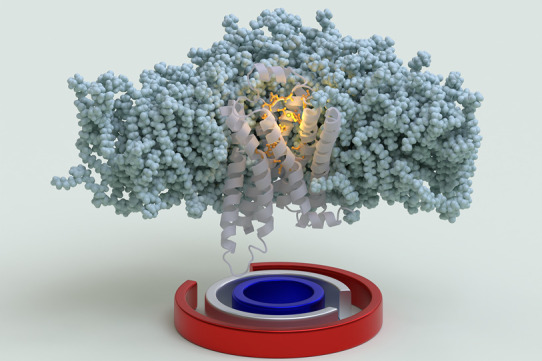

## Introduction

G-protein-coupled receptors (GPCRs) are one of the largest and most versatile families of membrane proteins, playing an integral role in transducing extracellular signals into cellular responses.^1,2^ They are involved in various physiological processes, including sensory perception, immune response, and neurotransmission.^3–5^ Owing to their central role in numerous signaling pathways, GPCRs have become prominent targets in the pharmaceutical industry, with approximately one-third of all marketed drugs acting on these receptors.^6–8^ In this regard, more than 1000 structures have been released on the Protein Data Bank (PDB), providing a wealth of structural information.^9–11^ However, experimental high-resolution descriptions of their activation mechanisms are difficult to obtain and have only been reported for a few receptors thanks to NMR,^12–14^ time-resolved X-ray crystallography, cryo-EM,^15,16^ single-molecule FRET,^17^ hydrogen–deuterium exchange mass spectrometry (HDX-MS),^18,19^ and double electron–electron resonance (DEER).^20,21^ Indeed, effectively capturing the full range of functional dynamics of a GPCR continues to be a significant challenge, crucial for the rational design of more effective and less toxic drugs.^8,22^

Among the proteins in this family, the beta-1 adrenergic receptor (ADRB1) plays a central role in regulating cardiovascular function and is a key therapeutic target in the treatment of heart diseases.^23,24^ Drugs such as metoprolol, bisoprolol, and atenolol act as ADRB1 antagonists (i.e., β-blockers) and are widely prescribed for hypertension, heart failure, and arrhythmias, making this receptor critical for managing cardiovascular health and preventing life-threatening events like myocardial infarction and stroke. Despite the pharmacological relevance of ADRB1, the mechanistic understanding of its activation remains incomplete. In particular, the coordinated role of exogenous factors—such as water molecules, sodium ions, and endogenous ligands—and elements like conserved microswitches in driving activation is still not fully understood.

To try to fill this gap, molecular dynamics (MD) simulations have proven invaluable in elucidating ligand recognition and receptor dynamics.^25–27^ Yet, conventional MD is limited by the timescales it can sample, often falling short of capturing the large-scale conformational transitions required for receptor activation.^22,28^ Enhanced sampling methods based on collective variables (CVs)—such as Metadynamics,^29,30^ Umbrella Sampling, and Gaussian accelerated MD^31^—have addressed this limitation to some extent.^32–35^ Nevertheless, defining optimal CVs that capture the relevant slow degrees of freedom remains nontrivial and often leads to convergence issues and high computational cost.^36,37^ For instance, Metadynamics was used to investigate the binding mode and affinity of adrenaline and noradrenaline toward ADRB1 and beta-2 adrenergic receptor (ADRB2)^38^ as well as the effect of various ligands on the activation energy landscape of ADRB2.^28,39^ Nevertheless, even with the Metadynamics approach, it is necessary to use system-specific collective variables and long sampling times, often combined with multiple replica algorithms, to converge the free energy landscapes associated with GPCRs. This means that, in most cases reported in the literature so far, only one calculation could be performed, and the sampling error was estimated by averaging the computed energy profiles over time. To overcome these challenges, we combined OneOPES^40^ with a set of tailored CVs specifically designed to capture the coupled dynamics of water networks,^41^ sodium ions,^42^ protonation states,^43^ and microswitch rearrangements during GPCR activation.^44^ OneOPES introduces a temperature gradient across replicas and accelerates several CVs simultaneously, enabling efficient convergence of complex free energy landscapes, even when CVs are suboptimal. While OneOPES has previously been applied to protein folding and ligand binding,^45–47^ this work presents its first application to the activation of a GPCR.

Using this approach, we studied the activation mechanism of ADRB1 in its apo- and adrenaline-bound states. The method provided reproducible and converged free energy landscapes with a limited number of replicas and manageable sampling times, capturing the impact of agonist binding, sodium ions in the conserved allosteric pocket, and protonation states of key residues. Additionally, we observed the rearrangement of a conserved intrahelical water network, which plays a central role in facilitating the structural transitions associated with receptor activation.^47^ These findings align with recent work highlighting the importance of water-mediated allosteric networks in GPCR function and design.^48–50^

Overall, our work offers a detailed and dynamic view of ADRB1 activation, bridging structural and energetic perspectives. The novel computational framework introduced here is efficient, reproducible, and applicable to other GPCRs and provides a foundation for rational drug design targeting receptor activation mechanisms.

## Computational Methods

### Systems Preparation

The apo- and holo-structures of ADRB1 were generated starting from PDB ID: 7BVQ and 7BTS,^38^ respectively. The apo structure (PDB ID: 7BVQ) encompasses only a portion of the whole human ADRB1 sequence, notably residues S^1.28^ to S^5.74^ and V^6.26^ to C^8.59^. In the experimental structure, the ends of the sequences S^5.74^ and V^6.26^ were artificially linked, resulting in a shorter intracellular loop 3 (ICL3) compared to that of the native sequence. Regarding the holo-structure, ICL3 is unresolved in PDB ID: 7BTS (from V^5.69^ to A^6.27^). For this reason, the missing patch was taken from 7BVQ and merged with the experimental structure. For both apo- and holo-ADRB1, the couples C^3.25^–C^45.50^ and C^45.43^–C^45.49^ were bound with a disulfide bridge. As described in previous studies,^38^ E^3.41^ was protonated because it directly contacts the phospholipid tails and predominantly exists in its neutral form.

The complexes thereby obtained were embedded into a simple phospholipid bilayer (POPC/CHL 80:20) using CHARMM-GUI^51^ and solvated with the TIP4PD water model (salinity of 0.15 M NaCl). The N-terminus and C-terminus of both ADRB1 structures were capped with acetyl and methylamino protecting groups, respectively. The DES-Amber force field was employed^52^ in the MD engine GROMACS 2023.^53^ Each simulation box underwent a thermalization cycle with decreasing time-dependent restraints on heavy atoms in order to relax unphysical bond lengths and retain the overall structure of the protein and the membrane components. All systems experienced the following protocol: 1 ns of NVT simulation followed by 1 ns of *NPT* simulation for each temperature, starting from 100 to 300 K with steps of 50 K. The integration step was set to 2 fs. Coulomb, and van der Waals interactions were cutoff at a distance of 1.0 nm. The particle-mesh-Ewald (PME) method was used to treat the long-range electrostatic interactions.^54^ The temperature was set at 300 K and controlled with the V-rescale thermostat,^55^ whereas the pressure was fixed at a reference value of 1 bar with the semi-isotropic C-rescale barostat.^56^ To generate the reference inactive apo-ADRB1 structure, we carried out a 500 ns unbiased MD simulation on the carazolol-less 7BVQ structure embedded in the POPC/CHL bilayer. For a detailed analysis, please refer to Figure S1.

### OneOPES MD Simulations

A CV-based enhanced sampling method was adopted to investigate the conformational change of apo- and holo-ADRB1. Notably, we employed the OneOPES sampling scheme,^40^ a derivative technique of the “On-the-fly probability enhanced sampling” (OPES) algorithm in its “Explore” flavor.^57^ In OneOPES, a replica-exchange framework of 8 independent trajectories is set up to ensure the exploration and convergence of the FES under investigation. Such replicas are divided into two groups, i.e., a convergence-dedicated replica (replica **0**) and seven exploratory trajectories (replicas **1–7**). They all share OPES Explore as the main sampling engine carried out on a set of leading CVs. Replicas **1–7** are progressively heated (up to 335 K), thanks to OPES Expanded (OPES MultiT, hereafter), to ease overcoming hidden degrees of freedom.^58^

All CV-based enhanced sampling methods require a careful choice of CVs to efficiently converge the free energy. In the case of GPCR activation, the main and auxiliary CVs have to cover the slow degrees of freedom linked to the GPCR activation, namely, the macroswitches (helix movement) and microswitches (conserved motifs that are known to switch from the inactive to the active state). As our main CV, we chose the “*PATH*” CV^59^ connecting the inactive to the inactive state because it has been shown by us and others to provide a fair approximation of the macro conformational changes for kinase and GPCR activation.^33,39,60^

Here, several steered MD simulations were used to generate trajectories connecting the inactive to active conformations of ADRB1. Of these, the trajectory with the lowest work was retained. A PATH CV was defined by selecting 13 equidistant protein conformations (i.e., milestones) along the selected trajectory. While this CV is able to drive the overall motion of the Cα atoms, full activation of GPCRs is largely driven by the rearrangements of some selected amino acids’ side-chains that go by the name of “*micro-switches*”.^61^ To this end, we selected the following three sets of microswitches to enhance the quality of the sampling.

- **PIF:** between the residues P^5.50^, I^3.40^, and F^6.44^, facilitating the movement of TM6;
- **DRY:** located at the cytoplasmic end of TM3, between D^3.49^, R^3.50^, and Y^3.51^;
- **NPxxY:** a sequence motif found in TM7, composed of N^7.49^, P^7.50^, and Y^7.53^.

In this study, the distances between the elements of these motifs were treated as individual CVs to effectively capture and encourage transitions between the active and inactive states of ADRB1.

The role of a sodium ion binding to a conserved sodium pocket and conserved intrahelical water networks are also well-known.^48,49,62^ In particular, the presence of water molecules bridging the Y^7.53^ of the NPxxY motif and Y^5.58^ has been reported to facilitate the receptor’s activation. For the movement of the sodium ion, we defined a PATH CV (see Supporting Information), while we included a “*Hydration*” CV between the side chains of Y^5.58^ and Y^7.53^ to sample the changes in the crucial water network.^63^ All of these auxiliary CVs have been implemented in the exploration-dedicated replicas. For a complete description of the atoms involved in the abovementioned CVs, please refer to Figures S2 and S7.

In this framework, the exchange frequency among the replicas was 5000 integration steps. Replicas **0–7** contain a common layer of OPES Explore with a bias of 100.0 kJ/mol and a PACE (i.e., frequency of bias deposition) of 50,000 steps. Moreover, replicas **1–7** underwent additional OPES Explore layers (OPES MultiCV) with a bias of 3.0 kJ/mol on the auxiliary CVs and a PACE of 100,000 steps. We selected the following temperature range, i.e., [300 K: 301 K] for replica **1**, [300 K: 303 K] for replica **2**, [300 K:306 K] for replica **3**, [300 K: 310 K] for replica **4**, [300 K: 317 K] for replica **5**, [300 K: 325 K] for replica **6**, and [300 K: 335 K] for replica **7**. For clarity, a full description of the different CVs and parameters used for the OneOPES simulations has been reported in Tables S1 and S2. To run the simulations, the MD engine GROMACS 2023 patched with PLUMED 2.9 was employed.^64,65^ Regarding the thermostat and the barostat, we used the same protocol described in the System preparation section.

To evaluate whether the selected set of CVs is sufficient to describe the activation mechanism, one practical approach is to monitor the progression of the PATH CV as a function of simulation time and applied bias (see Figures S4 and S5). This visualization provides direct insight into the convergence and sampling efficiency. When the bias projected onto the PATH CV stabilizes (particularly after ∼150 ns, as indicated by the color gradient), it suggests that the relevant conformational space has been adequately sampled. This is typically reflected in the low variability of the free energy profiles obtained from independent OneOPES simulations. What happens to the convergence of the free energy landscape when we miss a crucially slow degree of freedom? In Figure S3, we show, for instance, the case of the free energy reconstructed without considering the hydration of conserved cavities. The plot of the PATH CV versus sampling time clearly shows that the sampling is suboptimal close to the active state. Consequently, the reconstructed FES will artificially penalize the active state, placing it at a significantly higher energy compared with the fully converged FES.

### Cluster Analysis

Cluster analyses on the MD trajectories were performed using GROMACS’s *gmx cluster* routine, using the *gromos* algorithm. An RMSD threshold value of 2.0 Å was selected considering the number of generated cluster families and the similarity of the protein conformations within a cluster family.

### Binding Interface Evaluation

To assess the interactions between the adrenaline and ADRB1 in the holo-ADRB1 MD simulations, we used the PLOT NA routine of the “Drug Discovery Tool” (DDT) to estimate the frequency of occurrence of contacts.^66^ To analyze the binding interfaces, we followed the approach of previous studies and set a neighboring cutoff value of 3.5 Å between the ligand and interacting residues.

## Results and Discussion

### Apo and Holo ADRB1 Simulations

The main objective of our study is to devise a robust and efficient enhanced sampling approach that could reproducibly reconstruct the free energy landscape associated with the activation of the beta-1 adrenergic receptor (ADRB1) in its apo form and its endogenous ligand (adrenaline) bound form. Each OneOPES simulation of ADRB1 was run independently three times using 8 replicas, with each independent simulation accumulating a total of 16.8 μ s of sampling for the apo and 21.6 μ s for the holo (see Figures S4 and S5 for additional details). We also repeated the simulations to quantify the effects of D^2.50^ and the presence of Na^+^ in the conserved sodium pocket.

To enhance the clarity of the discussion, in the following, we present various 1D and 2D projections. For the first and main projection, we use an overall “*Conformational Change*” (CC) CV, which is a combination of RMSD from active and inactive structures that is able to track even subtle differences in ADRB1’s plasticity along the functional dynamics (see Figure S6a,b for additional details). Reweighting the accumulated bias potential from the 3 independent simulations onto this CV yields 3 1D FES. In Figure 1c, the curves are shown together with the average and the error bands around the average obtained from the independent simulations. In Figure 1d, the convergence of the Δ*G* between the active and inactive states is shown. After ∼700 ns, all three independent simulations converge to a value of 13 ± 1 kcal/mol. We stress the importance of testing the convergence of the free energy profiles and computing the error bars from independent simulations. This is far from trivial and, so far, due to the significant computational cost, most of the free energy landscapes reported in the literature for GPCR activation, including our own,^28,32^ have been obtained from a single run and the error bars from block analysis rather than from multiple independent simulations.

**Figure 1:**
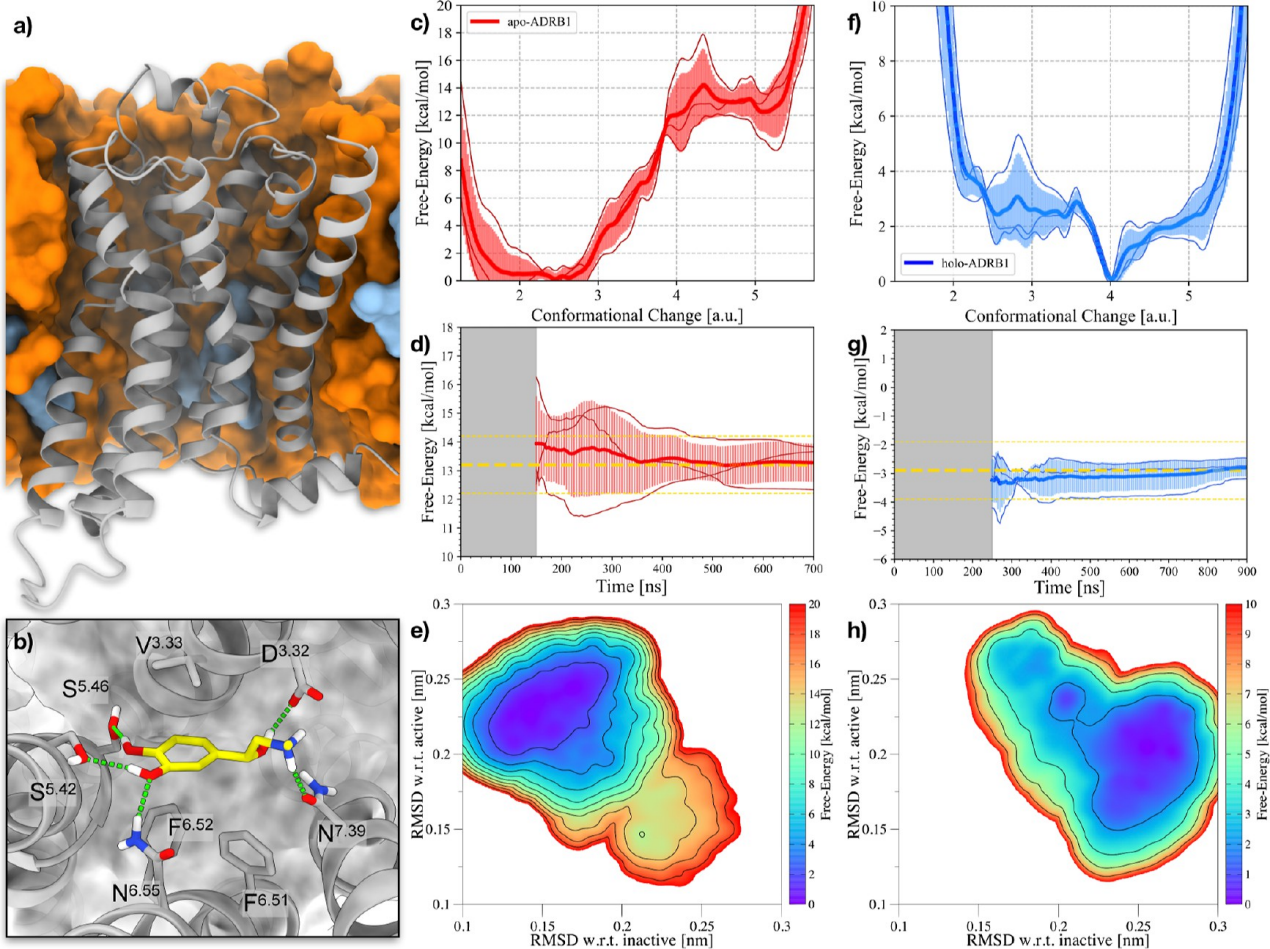
Results of the OneOPES simulations on the apo- and holo-ADRB1 systems. (a) ADRB1 embedded into a POPC/CHL (80:20) model membrane. ADRB1 is colored in gray, whereas POPC and CHL are depicted in orange and cyan, respectively. (b) Binding mode of adrenaline (in yellow) bound to ADRB1. (c) 1D FES as a function of the Conformational Change CV for the apo-ADRB1 systems. (d) Free-energy difference between inactive and active states in the apo-ADRB1 systems over time. In (c,d), the red solid line represents the average of the three replicas, while the transparent red area illustrates the standard deviation. (e) Average 2D FES concerning the RMSD of both the inactive and active structures for the apo-ADRB1 systems. (f) 1D FES as a function of the Conformational Change CV for the holo-ADRB1 systems. (g) Free-energy difference between inactive and active states in the holo-ADRB1 systems over time. In (f,g), the blue solid line represents the average of the three replicas, while the transparent blue area illustrates the standard deviation. (h) Average 2D FES with respect to the RMSD of both the inactive and active structures for the holo-ADRB1 systems. In (d,g), the average value is shown as a yellow dashed line. Error bars of 1 kcal/mol appear as yellow dashed lines. In (e,h), isolines are drawn every 2 kcal/mol.

The free energy profile computed for the apo-ADRB1 is in qualitative agreement with the scheme proposed by Weis and Kobilka^67^ and by Wu et al. based on NMR measurements^13^ and reveals the presence of four distinct regions: a broader minimum at CC = 2–3, containing two subminima corresponding to the inactive, ionic lock closed (CC = 2) and inactive ionic lock open states (CC = 3), respectively. These two states can be clearly distinguished in the 2D FE projection shown in Figure 2b. A high-energy minimum at CC = 5 corresponds to the active state, while the shoulder at CC = 4 is an intermediate state along the activation pathway that, based on the status of the microswitches, was identified as the preactive state (see Figure 1b). The free-energy difference between the inactive and active states is consistently determined from the 3 independent OneOPES simulations to be 13 ± 1 kcal/mol. This value is obtained already after 150 ns of simulation and remains stable until the end of the simulations, providing a quantitative measure of the energetic cost associated with ADRB1 activation (Figure 1d).

**Figure 2:**
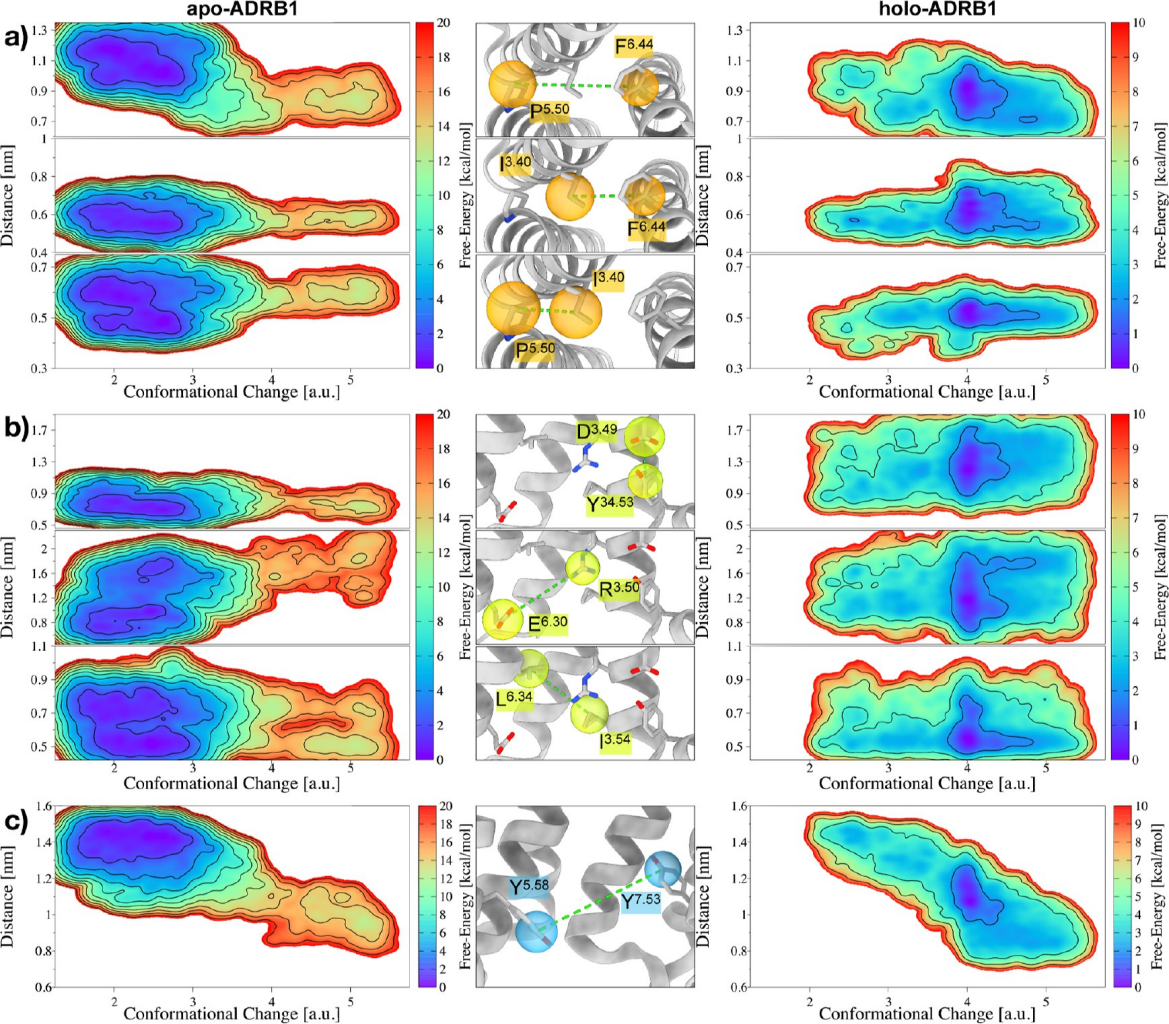
Analyses of ADRB1’s microswitches during its apo- and ligand-induced activation. (a) 2D FES as a function of the Conformational Change CV and the respective Distance CVs for the residues of the PIF motif (P^5.50^–F^6.44^, I^3.40^–F^6.44^, and P^5.50^–I^3.40^). (b) 2D FES as a function of the Conformational Change CV for the DRY motif (D^3.49^–Y^34.53^), the ionic lock (R^3.50^–E^6.30^), and an additional hydrophobic contact in the proximity of the DRY motif (I^3.54^–L^6.34^). (c) 2D FES as a function of the Conformational Change CV for the YY motif (Y^5.58^–Y^7.53^). On the left side of (a–c), we reported the average 2D FES of the 3 apo-ADRB1 OneOPES simulations, while on the right side, the average 2D FES of the 3 holo-ADRB1 OneOPES simulations. Isolines are drawn every 2 kcal/mol.

Our results agree with the mechanism deduced from NMR measurements,^12^ in particular with respect to the presence of the intermediate (preactive) state. Our calculated free energy difference for the activation of the apo WT receptor cannot be directly compared with NMR results because, as expected from the high free energy differences, the population of active states is too small to be measured in the experiments. Furthermore, NMR experiments were conducted on a thermostabilized mutant of turkey ADRB1 in the presence of isoprenaline, a full agonist closely related to adrenaline. This agonist promotes receptor activation, helping to stabilize the intermediate- and active-like conformations. In addition, the effect of the reported protonation of the conserved residue D^2.50^, which greatly reduces the free energy difference, must be taken into account (see, e.g., Figure 5c).

In the following, the 2D free energy projections presented are computed as an average of the FES from three independent simulations. Starting from the apo-ADRB1, we reweighted the accumulated bias potential on a 2D space defined by the RMSD with respect to both inactive and active ADRB1 structures (see Figure 1e). This representation enables the identification of a minimum-energy activation pathway, delineating the rearrangement ADRB1 must undergo for its activation. We repeated our OneOPES simulations on the holo-ADRB1 system to investigate the impact of a natural agonist, adrenaline, on receptor activation (Figure 1b). The 1D FES profile obtained by reprojecting the bias potential onto the CC CV shows a deep minimum at CC = 4, aligning with the previously identified intermediate state (see Figure 1f). As expected from NMR measurements, the presence of adrenaline stabilizes this conformational intermediate,^12^ effectively lowering the energetic cost toward activation, which, over the simulation time, we estimated in a free-energy difference of −3 ± 1 kcal/mol (see Figure 1g). The Δ*G* to the fully active state, which, as in the apo receptor, is found around CC = 5, is less than 2 kcal/mol. Also in this case, the relative population of the two states is significantly influenced by the protonation of D^2.50^, which shifts the equilibrium of the adrenaline-bound receptor toward the fully active state (Figure 6 for further details).

To properly discern the features of the adrenaline-induced preactive state, we extracted and analyzed the whole conformation pool of structures located at CC = 4. A cluster analysis on adrenaline’s binding mode revealed the presence of a single largely populated cluster, whose position in ADRB1’s orthosteric binding site slightly differs with respect to the crystallographic active state. As shown in Figure S6c,d, adrenaline is shifted toward TM5 in the intermediate state, a rearrangement that, while preserving most of the ligand-GPCR interaction network, disfavors the engagement of N^6.55^ with adrenaline’s hydroxy groups. Conversely, N^6.55^ tends to form a H-bond with N^7.39^’s side-chain, which also preserves its interaction with adrenaline’s amine. The identification of a novel intermediate structure of ADRB1 where adrenaline preferentially binds may have significant implications for drug discovery and rational design of potent ADRB1 agonists. This intermediate state represents a previously uncharacterized conformation that could serve as a valuable target for structure-based drug design approaches, such as molecular docking and virtual screening. To encourage further exploration and aid drug design efforts, we provide the atomic coordinates of this ADRB1 intermediate structure as part of the Supporting Information.

The 2D FES, built again using RMSD values relative to the inactive and active ADRB1 structures, shows a significant shift of the activation pathway toward regions of the free-energy landscape that were previously less explored in apo-ADRB1 (Figure 1h). Thus, our results provide evidence that adrenaline changes the activation dynamics, suggesting in turn an at least partially induced-fit (active state induction) mechanism where adrenaline binding facilitates the receptor’s transition toward active-like conformations. This observation, if confirmed by further simulations and kinetic experiments, might contribute to the ongoing debate in the GPCR field regarding whether agonists exert their effects through *conformational selection* or *induced-fit mechanisms*.^22^

### Modulation of ADRB1’s Microswitches

When we compare the active and inactive structures of a receptor, we see more localized changes in the specific regions. These “microswitch” changes involve events like specific residue-to-residue contacts and side-chain rotations. Of the more than 90 state determinant microswitches reported across different GPCR classes, only 4 seem to be shared among all the classes.^22^ Among the most critical microswitches involved in ADRB1 activation, the PIF motif (i.e., I^3.40^, P^5.50^, and F^6.44^) plays a fundamental role in linking ligand binding to intracellular conformational changes (see Figure 2a). In the apo-ADRB1 simulations, we observe a progressive reduction in the P^5.50^–F^6.44^ distance, shrinking from 1.3 nm in the inactive state to approximately 0.9 nm in the active state, marking a structural rearrangement that accompanies receptor activation. Conversely, in the holo-ADRB1 simulations, the P^5.50^–F^6.44^ distance oscillates stably around 0.9 nm throughout the whole Conformational Change CV, suggesting that the presence of adrenaline preconfigures the PIF motif into an activation-prone conformation. A similar trend is observed in the P^5.50^–I^3.40^ distance, which fluctuates between 0.4 and 0.7 nm in apo-ADRB1, reflecting the greater conformational flexibility of the receptor in the absence of the agonist. Instead, in the holo-ADRB1 simulations, this distance remains stably locked at 0.5 nm, even in conformations that correspond to the inactive state. These observations are consistent with an adrenaline-facilitated shift of ADRB1’s conformational equilibrium toward an active-like state en route to a full activation. This motif, together with the W^6.48^, was also reported to be crucial for the transmission of the allosteric signal from the orthosteric binding site to the effector binding site by Wu et al. based on NMR data.^13^ They even introduce the term “*xWIPF3*” to collectively indicate the motion of the two microswitches, as they appear to move in concert.

In addition to the PIF motif, another set of key microswitches that play a crucial role in ADRB1 activation is the DRY motif, consisting of D^3.49^, R^3.50^, and Y^3.51^ (see Figure 2b). This conserved triad is involved with the surrounding amino acids in stabilizing the inactive state of GPCRs, and it undergoes a significant rearrangement during receptor activation. Our analysis reveals that in the apo-ADRB1 simulations, the D^3.49^–Y^34.53^ hydrogen bond remains consistently engaged throughout the entire range of the Conformational Change CV. However, in the holo-ADRB1 simulations, the D^3.49^–Y^34.53^ distance becomes significantly more dynamic, fluctuating across a broad range of values between 0.9 and 1.7 nm. This increased mobility suggests that the presence of adrenaline weakens the constraints imposed by the DRY motif, allowing for a more flexible conformational landscape and facilitating the transition toward activation.

A similar trend is observed in the ionic lock, a crucial interaction between R^3.50^ and E^6.30^ that plays a well-established role in GPCR activation by stabilizing the inactive conformation. In the apo-ADRB1 simulations, the R^3.50^–E^6.30^ distance follows a well-defined activation pathway: in the inactive state, the salt bridge between R^3.50^ and E^6.30^ is broken, as displayed in the minimum at CC ∼ 3 and distance ∼ 1.6 nm. This is a prerequisite for subsequent conformational rearrangements, allowing ADRB1 to transition to its active state. However, in the holo-ADRB1 simulations, the R^3.50^–E^6.30^ interaction is far more mobile, displaying a broader distribution of values rather than following a single, well-defined reaction coordinate. Once again, the collected data suggest that the presence of adrenaline disrupts the tight structural constraints of the inactive state, increasing the receptor’s conformational plasticity and promoting alternative activation pathways.

Lastly, another crucial microswitch that plays a fundamental role in ADRB1 activation is the YY motif, involving Y^5.58^ and Y^7.53^ on the intracellular side of the receptor. This motif is a well-known conserved structural element in class A GPCRs, often implicated in the stabilization of receptor conformations and in the allosteric regulation of intracellular signaling. As displayed in Figure 2c, our analysis reveals that the evolution of the Y^5.58^–Y^7.53^ distance remains largely unperturbed by the presence of adrenaline, hinting that this specific interaction follows a similar activation pathway in both apo- and holo-ADRB1 simulations, aside from the shift of the deepest minimum in the 2D FES from CC ∼ 2.5 toward CC ∼ 4. The collected data may suggest that the YY motif does not exhibit significant structural alterations upon adrenaline binding. Nevertheless, it is important to stress that the YY motif is intrinsically linked to the hydration dynamics of ADRB1’s intracellular cavity. The Y^5.58^–Y^7.53^ interaction plays a pivotal role in controlling the influx and organization of water molecules, which, in turn, can significantly impact receptor activation by modulating key conformational transitions.^12,68^ In the pursuit of fully understanding the mechanistic effects of adrenaline binding, a comprehensive assessment of the hydration dynamics within ADRB1 has been carried out and is discussed in the subsequent sections of the manuscript.

### Hydration of ADRB1’s Intracellular Side

Changes in conserved water-mediated allosteric networks have been reported to play a crucial role in GPCR activation and signal transduction, including in ADRB1.^12,48,49,68^ We analyzed the water occupancy and dynamics in both the apo- and adrenaline-bound states emerging from our OneOPES simulations, focusing on the intracellular side, where conformational changes critical for G-protein coupling occur. To this end, we reweighted the accumulated bias potentials on a 2D FES function of the Conformational Change CV and, in this case, the “Hydration” CV, defined by the position of a dummy atom located between residues Y^5.58^ and Y^7.53^ (for additional details, please refer to the “Computational Methods” section and Figure S2). This approach allowed us to quantify the relationship between hydration dynamics and ADRB1’s conformational transitions, providing valuable insights into the structural features of the conformational ensembles associated with each basin of the CC against the hydration FES.

Regarding the data collected on the apo-ADRB1 simulations, we observed the presence of a single well-defined free-energy minimum at CC ∼ 5 and a Hydration value in the ∼0.5 to 1.5 range, as reported in Figure 3a. The analysis of the conformation pool corresponding to this basin let us identify transient 1–2 water molecules occupying the intracellular cavity. Such water molecules primarily interact with residues Y^5.58^ and Y^7.53^ of the YY-motif, forming short chains of hydrogen bonds between the two tyrosines mediated by water molecules. Conversely, the adrenaline-bound form exhibited a higher and more variable hydration pattern (e.g., up to 6 molecules), suggesting that ligand binding stabilizes a more hydrated environment (see Figure 3b). Indeed, by extracting the conformations belonging to the lowest basin (i.e., CC ∼ 4 and hydration ∼ 2.5), it was possible to observe a variety of water chains between Y^5.58^ and Y^7.53^, with the most frequent network being composed of 3 and 4 water molecules.

**Figure 3:**
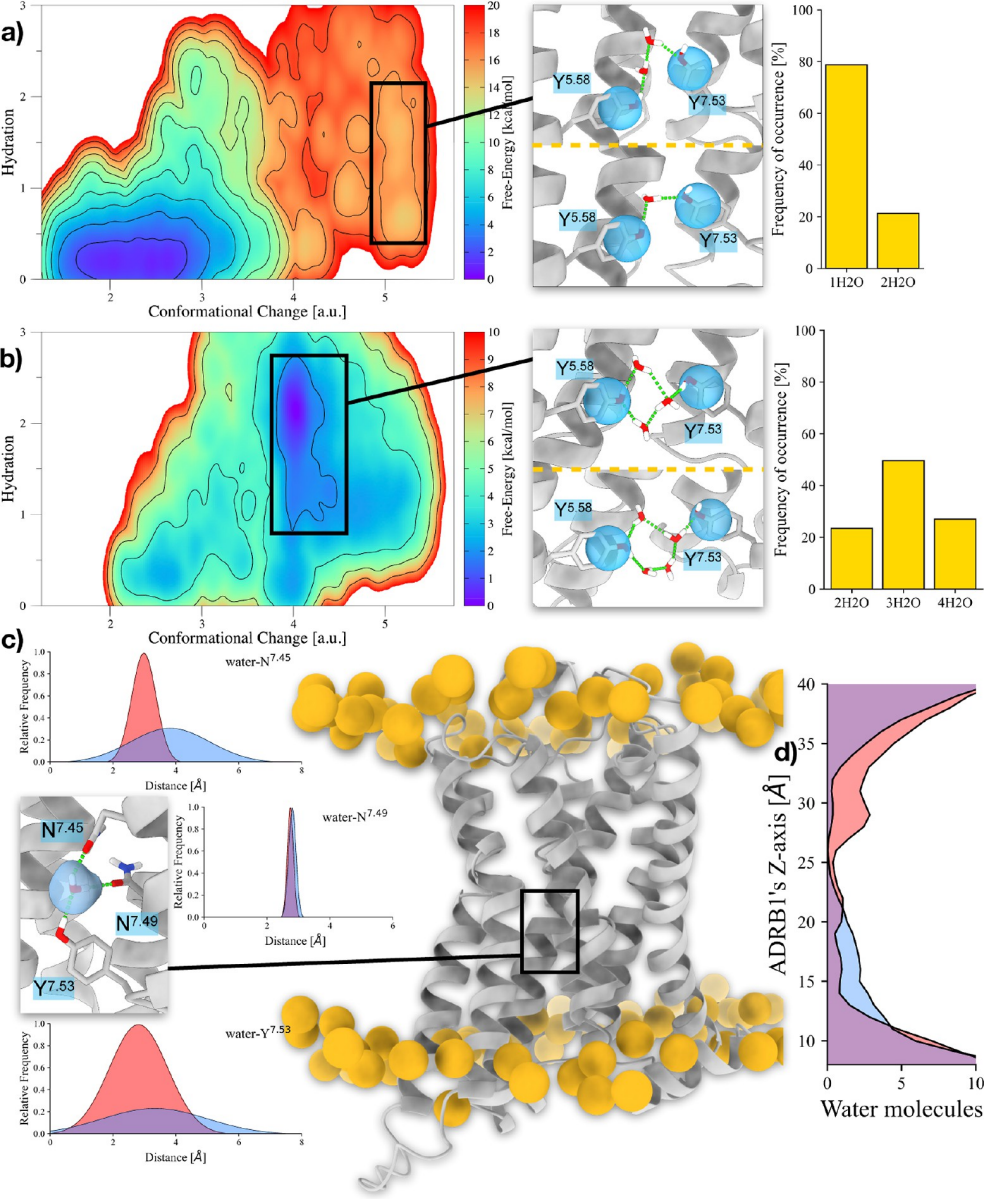
Differences in the internal hydration sites in apo- and holo-ADRB1. (a,b) 2D FES associated with the hydration of (a) apo- and (b) holo-ADRB1’s YY-motif during the GPCR activation. On the right side, insets displaying the water molecules connecting Y^5.58^ and Y^7.53^ and the relative frequency of each hydration state in the selected basin. (c) Average distances of N^7.45^ and N^7.53^ with respect to the closest water molecule to N^7.49^ in the apo- and holo-ADRB1 active basins. The image on the right depicts ADRB1 and the phosphorus atoms in the surrounding membrane. The inset displays the water molecule caged between N^7.45^, N^7.49^, and Y^7.53^. d) Distribution of water molecules along ADRB1’s *z*-axis. Values coming from apo- and holo-ADRB1’s active basins are colored in transparent red and blue, respectively.

To understand the underlying reason behind the differing hydration levels induced by adrenaline binding, we recognized that a purely numerical evaluation of the free-energy differences between the apo and holo conformations of ADRB1 would not be comprehensive. While the 2D FES provided critical insights into the hydration landscape, inspecting the structural impact of ligand binding in greater detail became imperative. We started by checking the overall cavity surrounding the YY-motif. This analysis let us observe the presence of structural water in the proximity of Y^7.53^. As depicted in Figure 3c, the conformational ensemble extrapolated from apo-ADRB1’s active basin is characterized by a rigid network established by N^7.45^’s and N^7.49^’s carboxamides with Y^7.53^’s hydroxyl, which all insist on a single water molecule. In holo-ADRB1, these interactions are much looser, leading to an increased level of hydration of the intracellular side of ADRB1 (see Figure 3d).

These analyses delivered a preliminary rationale for the differences in the intracellular activity between apo- and adrenaline-bound receptors yet do not clarify the consequences of ligand binding on ADRB1. So, we sought to reconstruct the cascade of structural rearrangements initiated by adrenaline at the extracellular side and how these propagate through the GPCR to influence the intracellular hydration environment. To achieve this goal, we compared the average pairwise distances of each residue pair in the apo- and holo-ADRB1 conformational pools. By mapping the shifts in residue pair distances, we could identify regions of the receptor undergoing significant rearrangements, which provided a mechanistic link between the extracellular binding event and its downstream effects on intracellular hydration.

As shown in Figure 4, the binding of adrenaline in ADRB1’s orthosteric site perturbs the hydrophobic packing between F^6.52^ and W^6.48^ (part of the xWIPF3 motif), closely stacked in apo-ADRB1’s active state. At the same time, the positively charged amine of adrenaline forms a salt bridge with D^3.32^, which in turn stabilizes the charge-enforced H-bonds with Y^7.43^. This first layer of interactions of adrenaline propagates onto the same amino acid, i.e., N^7.45^. Indeed, in apo-ADRB1’s active state, N^7.45^’s amide is stabilized by an H-bond with W^6.48^’s side-chain. Conversely, the displacement of W^6.48^ enhances the flexibility of N^7.45^ in holo-ADRB1, with the latter residue more prone to bind to G^7.42^’s carbonyl oxygen, in closer proximity due to Y^7.43^’s aforementioned binding with D^3.32^.

**Figure 4:**
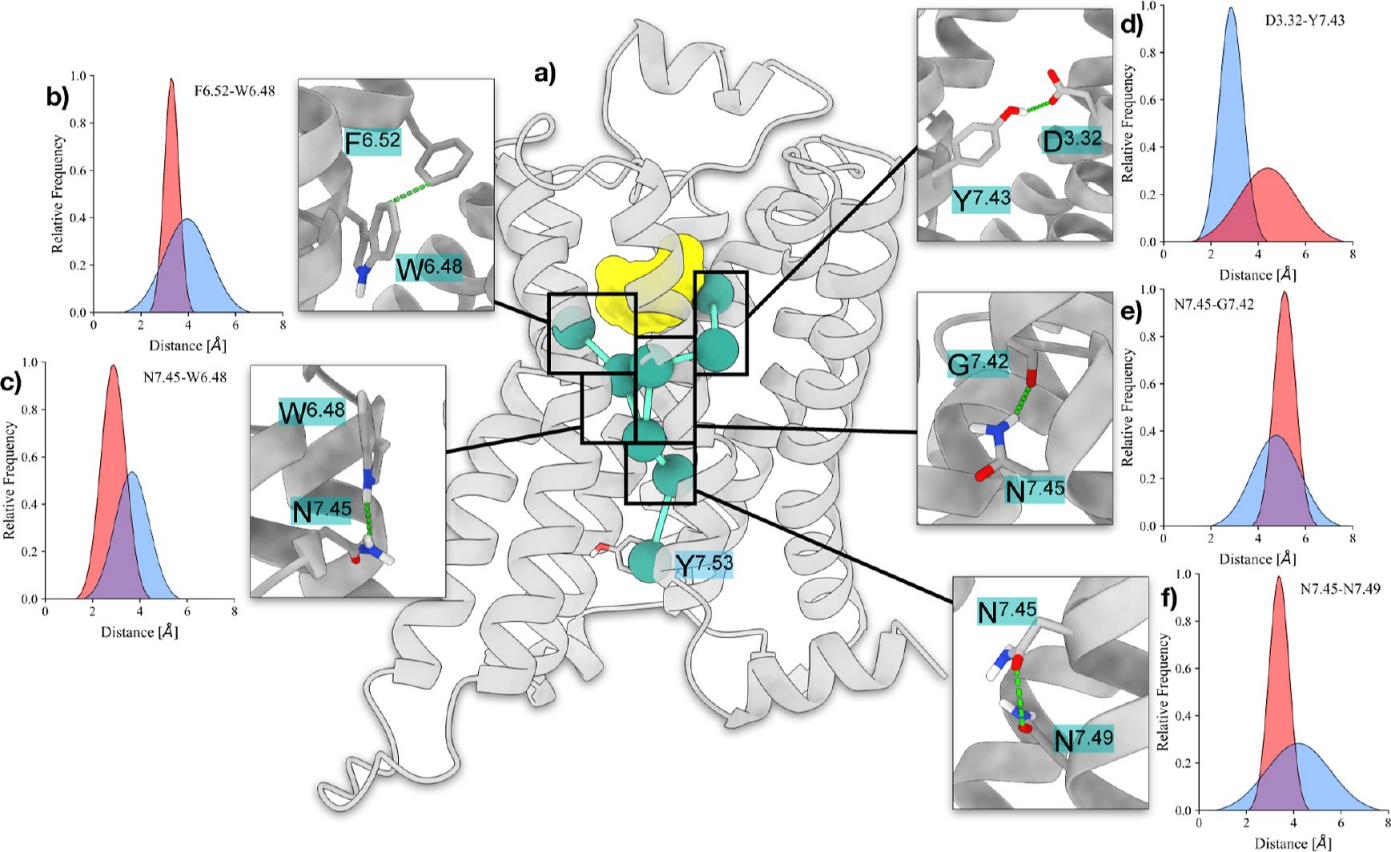
Schematic depiction of the effects of adrenaline binding at ADRB1 and the resulting propagation of structural changes through the receptor. (a) Network of intraprotein contacts that initiates a series of conformational changes extending to the intracellular side upon adrenaline (yellow) binding. (b–f) Average pairwise distances between the pair of residues F^6.52^–W^6.48^ (b), N^7.45^–W^6.48^ (c), D^3.32^–Y^7.43^ (d), N^7.45^–G^7.42^ (e), and N^7.45^–N^7.49^ (f). Values collected from apo- and holo-ADRB1’s active basins are colored in transparent red and blue, respectively.

Overall, this cascade of events led to an increase of the distance between N^7.45^ and N^7.49^, destabilizing the water-mediated interaction established between N^7.45^, N^7.49^, and Y^7.53^ presented in the previous paragraph and favoring the entrance of additional water molecules in the intracellular side of ADRB1. These suggestions are further supported by the work of Chen et al.,^49^ who, during the preparation of this manuscript, engineered highly signaling GPCRs from scratch by optimizing their intraprotein water network (focusing on residues W^6.48^, N^7.45^, N^7.49^, and Y^7.53^). Their findings align with ours, demonstrating that the interaction between water molecules and polar residues can facilitate movements in GPCRs that are prone to activation.

### Allosteric Modulation in the Sodium Binding Site

The sodium binding site is a conserved structural feature of class A GPCRs in many of which sodium has a well-established role as negative allosteric modulator.^62,69,70^ In ADRB1, as well as other class A GPCRs, this site is located near the conserved D^2.50^ residue,^71^ which serves as a primary coordinating partner for sodium.^72,73^ Notably, D^2.50^ is also known to become protonated during receptor activation, a mechanism that stabilizes the active state and prevents sodium coordination.^43,74^ To investigate these two effects (namely, the impact of sodium binding and D^2.50^ protonation on ADRB1 activation energy landscape), we carried out three novel sets of OneOPES simulations, each consisting of three independent replicas: apo-ADRB1-Na^+^, where we explicitly modeled sodium binding, apo-ADRB1-ASPH, where D^2.50^ was protonated, and holo-ADRB1-ASPH, where D^2.50^ is protonated in the presence of the adrenaline in the orthosteric binding site.

For apo-ADRB1-Na^+^, we extended our OneOPES sampling strategy to include a dedicated set of CVs that capture Na^+^ movement across the orthosteric cavity of the receptor (see Figure 5a and Table S2). Specifically, we introduced a *NaPATH* CV to track Na^+^’s motion from the extracellular side to the sodium binding site and a *NaWater* CV to monitor the hydration of sodium binding site itself (see Figure S6). Reweighting the accumulated bias potential onto the Conformational Change CV and averaging the results over the three replicas, we observed that sodium binding markedly stabilizes the inactive state of ADRB1.

**Figure 5:**
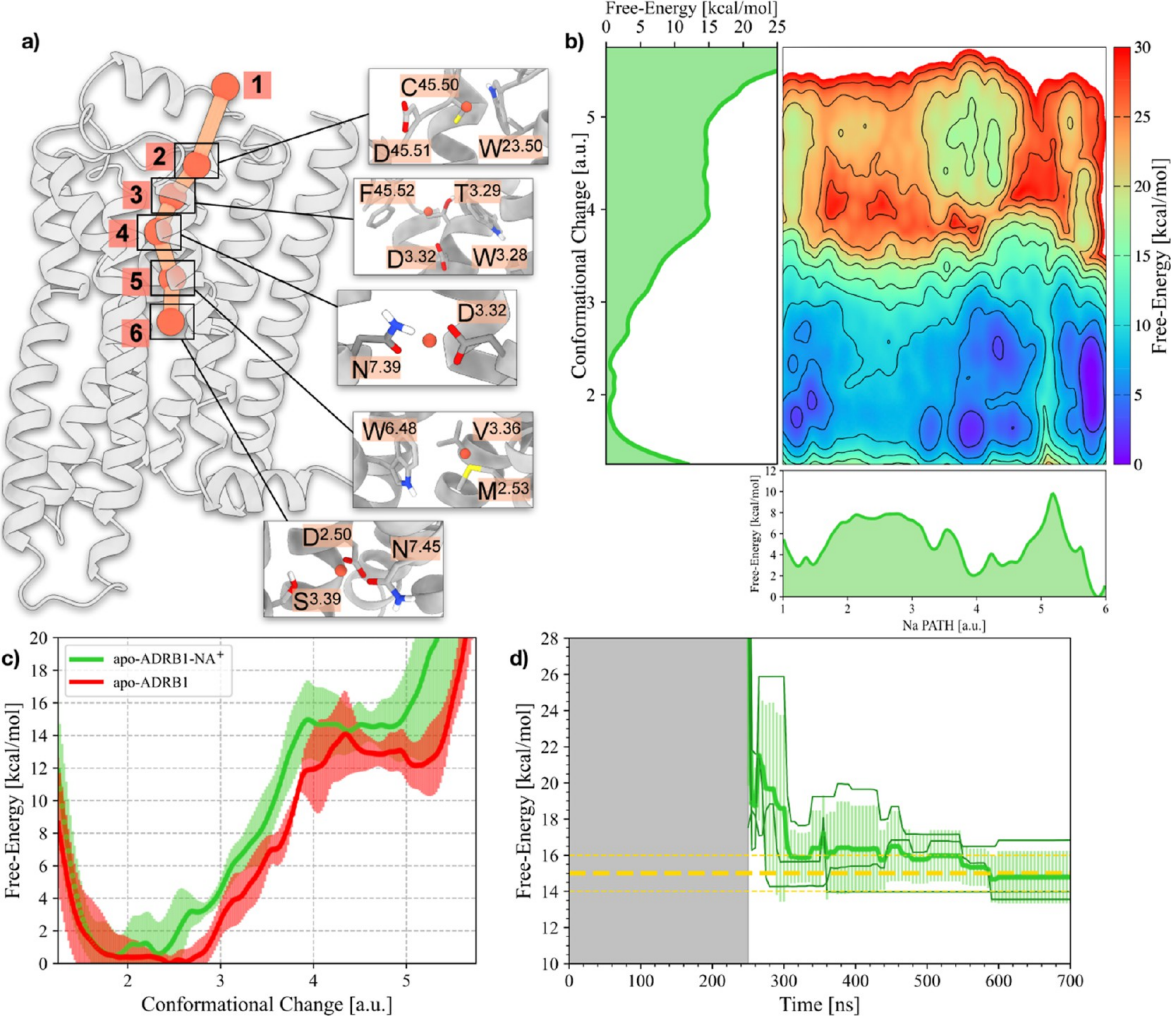
Allosteric modulation of the Na^+^ binding cavity. (a) Schematic representation of the Na^+^ translocation pathway (NaPATH CV) within ADRB1. Milestones along the pathway are indicated as orange spheres and shown with inset enlargements for clarity. (b) 2D FES illustrating the relationship between Na PATH (*x*-axis) and ADRB1’s Conformational Change CV (*y*-axis). 1D FES projections for the Na PATH and Conformational Change are also shown, providing insights into the energetic landscapes of these processes. (c) 1D FES as a function of the Conformational Change CV for the apo-ADRB1 and apo-ADRB1-NA^+^ systems. (d) Free-energy difference between inactive and active states in the apo-ADRB1-NA^+^ system over time. The average value is shown as a yellow dashed line. Error bars of 1 kcal/mol appear as yellow dashed lines.

Notably, we identified a significant energy barrier around milestone 5 of the NaPATH CV, where the ion must overcome a tightly packed hydrophobic cluster formed by M^2.53^, V^3.36^, and W^6.48^ (see Figure 5b). This suggests that sodium entry is highly restricted at this point, reinforcing the notion that hydration dynamics and local structural rearrangements play important roles in sodium accessibility. Our free-energy calculations also indicate that ADRB1 activation is significantly disfavored in the presence of sodium, with a Δ*G* of activation of 15 ± 1 kcal/mol, in agreement with experimental results (e.g., ^19^F NMR, ^23^Na NMR^72^), which show that sodium acts as a negative allosteric modulator by stabilizing the receptor in its inactive conformation (see Figure 5c,d).

We recomputed the activation free energy landscape of the apo-ADRB1-ASPH and holo-ADRB1-ASPH simulations, where D^2.50^ was protonated. For both systems, the results revealed a significant population shift: the neutralization of D^2.50^ shifts the receptor toward the preactive and active state (see Figure 6a,b). In particular, for apo-ADRB1-ASPH, the Δ*G* of activation is reduced to only 3 ± 1 kcal/mol, making the preactive and active states very close in energy (see Figure 6c). For holo-ADRB1-ASPH, instead the active state (CC ∼ 5) is the most stable conformation, suggesting a synergistic effect between agonist binding and D^2.50^ protonation. This effectively pushes the receptor toward the active ensemble of ADRB1, which is predicted to be −5 ± 1 kcal/mol more stable than the inactive state (see Figure 6d). A similar picture is obtained by reweighting the accumulated bias potentials on the 2D FES defined by the RMSD with respect to both inactive and active ADRB1 structures (see Figure 6e,f). With respect to Figure 1e,h, it is possible to see the shift of the free-energy landscapes and their minima toward active structure-like conformational pools. These findings align with the known role of D^2.50^ protonation in receptor activation, further confirming that sodium binding and D^2.50^ protonation have opposite effects on the conformational equilibrium of ADRB1.

**Figure 6:**
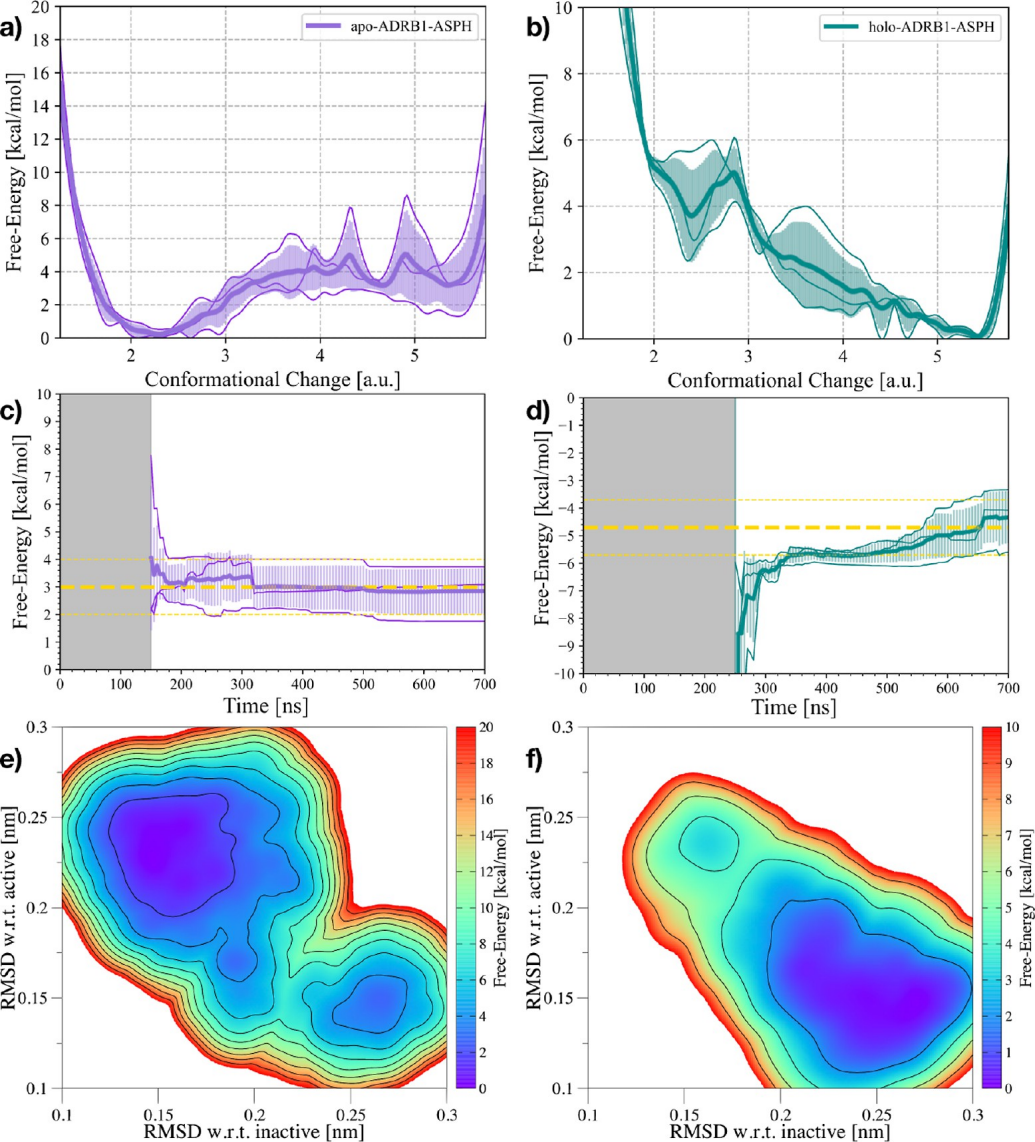
Results of the OneOPES simulations on the apo- and holo-ADRB1-ASPH systems. (a,b) 1D FES as a function of the Conformational Change CV for the apo-ADRB1-ASPH (a) and holo-ADRB1-ASPH (b) systems. (c,d) Free-energy difference between inactive and active states in the apo-ADRB1-ASPH (c) and holo-ADRB1-ASPH (d) systems over time. In (a,c), the purple solid line represents the average of the three replicas, while the transparent purple area illustrates the standard deviation. In (b,c), the teal solid line represents the average of the three replicas, while the transparent teal area illustrates the standard deviation. The average value is shown as a yellow dashed line. Error bars of 1 kcal/mol appear as yellow dashed lines. (e,f) Average 2D FES with respect to the RMSD of both the inactive and active structures for the apo-ADRB1-ASPH (e) and holo-ADRB1-ASPH (f) systems.

In physiological conditions, we can hypothesize that D^2.50^ is initially protonated, facilitating the dynamical equilibrium with preactive and even active states. In these states, Na^+^ has a lower barrier to enter the conserved sodium pocket and, once there, it “locks” the receptor in an inactive state until an agonist binds to the orthosteric site. In this regard, previous studies suggested that the presence of an agonist may then facilitate sodium egress toward the intracellular environment, potentially cutting down the free-energy cost associated with the full activation process.^72^ For additional details regarding the apo-ADRB1-ASPH and holo-ADRB1-ASPH simulations, please refer to Figures S7 and S8.

## Conclusions

The ADRB1 receptor is an important pharmacological target playing a crucial role in cardiovascular regulation.^75–77^ Our study aimed to leverage a novel, efficient, and reproducible enhanced sampling technique, OneOPES, and to develop tailored collective variables to characterize the functional dynamics of class A GPCRs such as ADRB1. Through its combination of replica exchange, thermal ramp, and multi-CVs, OneOPES has shown its ability to provide fully converged and reproducible results in a shorter simulation time compared to traditional methods, making it a powerful tool for studying GPCR activation.

The consistency across three independent, multireplica simulations for each state (apo, holo, and protonated) confirms the reproducibility and robustness of our results. Previously, high computational costs necessitated approximating error bars on free energy profiles with methods, such as block analysis. In contrast, the efficiency of our approach enables multiple repetitions, allowing us to directly calculate the error bars for these complex systems for the first time. By doing so, we address a key uncertainty in MD studies of GPCRs (i.e., the convergence of the computed free energy landscape), pave the way to investigate other sources of uncertainty, such as the force field, and eventually validate the results with quantitative experimental data.

We employed the DES-Amber force field for the protein and GAFF2 for the ligand. DES-Amber was selected based on favorable comparisons with NMR data,^52,78^ while GAFF2 was chosen for its proven ability to reproduce binding free energies.^46^ We acknowledge that the choice of force field significantly impacts the calculated populations of intermediate and end-point states. For example, published free energy profiles for the related ADRB2 receptor are qualitatively similar to our results but differ quantitatively depending on the force field used.^28,39^ While some of this discrepancy can be attributed to ADRB2’s higher basal activity, the force field itself has been shown to be a major source of quantitative variation. Thus, in follow-up studies, it would be interesting to use the proposed OneOPES strategy to converge the activation free energy landscape of ADRB1 and other GPCRs with different force fields and compare the results with quantitative experiments.

In this respect, as discussed above, the computed free energy profiles for the apo and holo receptors cannot unfortunately be directly compared to the available high-resolution NMR data of refs 12, 13, 68, 79, and 80 due to differences in the amino acid sequence and ligands, as well as the introduction of thermostabilizing mutations and the use of detergents in the experiments. It is also worth noting that in our simulations, we employed a POPC:cholesterol mixture, which serves as a biophysically relevant yet simplified representation of the plasma membrane. As shown in previous studies, the presence of cholesterol in the membrane and different membrane composition can significantly alter GPCR behavior and activation thermodynamics.^13,81^ Nevertheless, qualitatively, our on-path preactive intermediate is fully consistent with NMR observations. The simulations also agree with the NMR observations on the importance of the xWIPF3 switch and of the conserved water network.

In addition to providing key insights into the receptor’s activation pathway, our study offers a comprehensive overview of ADRB1 activation in both the apo- and adrenaline-bound states. Our detailed analysis of the role of water in the activation mechanism is in line with previous reports^48,49^ and complements them with unprecedented details of the step-by-step role of water-mediated allosteric networks along the activation reaction coordinate. Moreover, the reorganization of internal hydration upon agonist binding—marked by the merging of the water pockets around D^2.50^ and Y^7.53^—mirrors the distinct hydration topologies observed between active and inactive GPCR structures in both simulation and experimental studies. These results reinforce the idea that dynamic water networks serve as conserved allosteric features to modulate receptor activation. Further emphasizing the relevance of our findings, Rangari et al. recently reported a CB1 agonist that exerts its effect by targeting the receptor’s sodium binding site,^50^ specifically occupying a cryptic hydrophobic hotspot that separates the sodium binding site from the orthosteric one. This further highlights the role of the sodium and water dynamics in modulating receptor activation. In this context, our dedicated *NaPATH* and *NaWater* CVs can provide powerful tools to capture these mechanistic events with high resolution. A key finding is that a continuous water channel forms between the D^2.50^ pocket and the cytosolic cavity only in the presence of adrenaline. This suggests that ligand binding directly influences the internal hydration network, possibly by selectively stabilizing specific intermediate states. This mechanistic insight leads to testable hypotheses about the interplay among hydration, microswitches, and ligand efficacy. These hypotheses could be validated experimentally through site-directed mutagenesis or water-sensitive spectroscopic techniques like NMR.

Future drug discovery efforts could leverage the proposed computational framework to predict the efficacy of potential ligands and suggest chemical modifications to make them more effective.

In this respect, OneOPES has the potential to accelerate the development of next-generation drugs that target ADRB1, paving the way for the design of more effective and selective therapies for cardiovascular diseases.

## Supplementary Material



## Notes

The input files to replicate all the simulations can be found on https://github.com/valeriorizzi/ADRB1_OneOPES and on PLUMED NEST^82^ https://www.plumed-nest.org/eggs/25/020/ Additional information on enhanced sampling simulations and CV building (PDF)
